# Supplementary material for: Phenotype-Specific Recalibration of MAVE Data Enables Repurposing of BAP1 Functional Assays for Küry-Isidor Syndrome
Source: medRxiv. 2026 May 21:2026.05.15.26352805. Preprint. [Version 1] doi: 10.64898/2026.05.15.26352805 (PMC13228737; doi:10.64898/2026.05.15.26352805)
Supplement: Supplementary file 1 [file NIHPP2026.05.15.26352805v1-supplement-4.pdf]

medRxiv preprint doi: https://doi.org/10.64898/2026.05.15.26352805; this version posted May 21, 2026. The copyright holder for this preprint
(which was not certified by peer review) is the author/funder, who has granted medRxiv a license to display the preprint in perpetuity.
It is made available under a CC-BY 4.0 International license .

Supplementary information

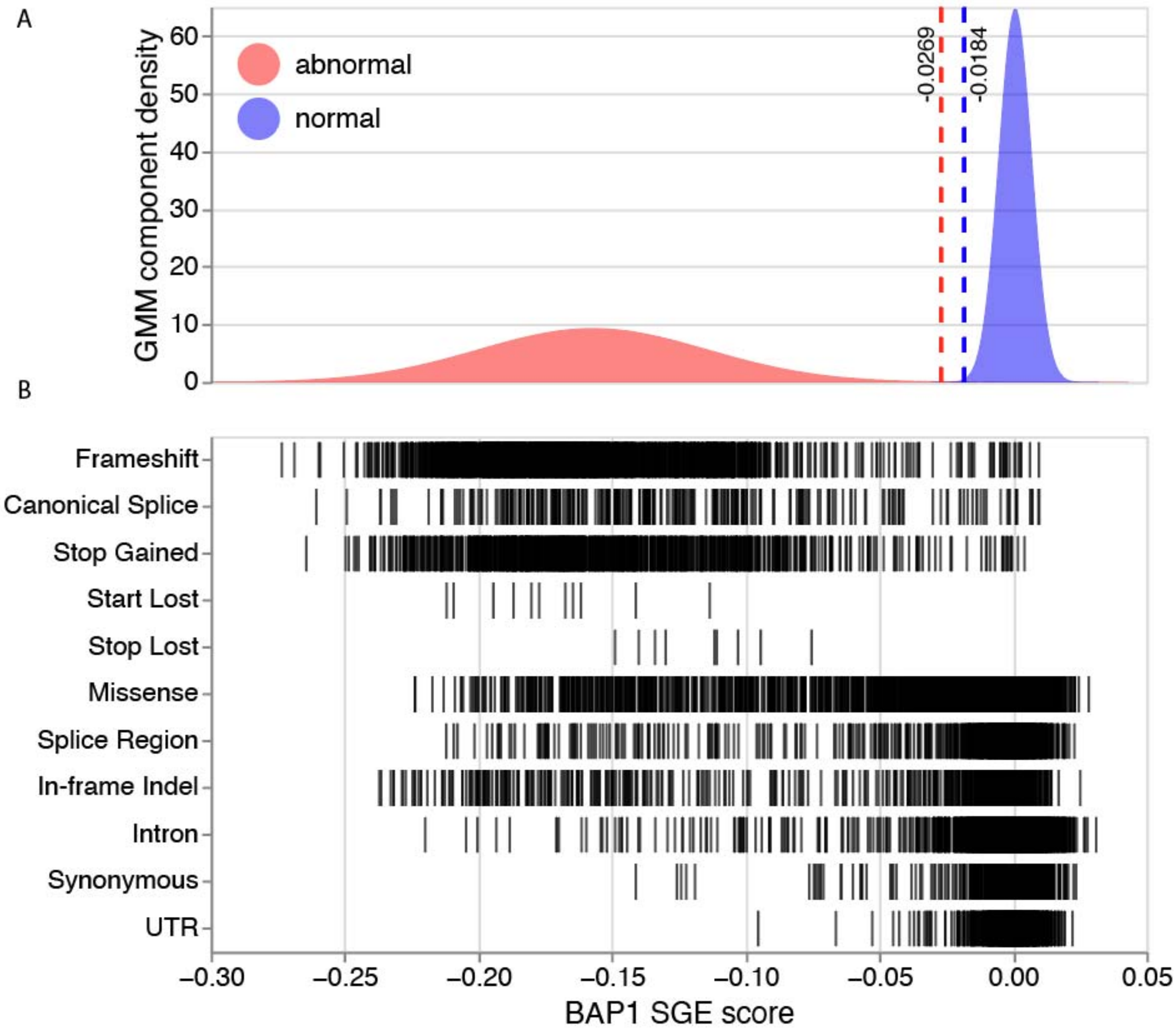

Supplemental Figure 1: Fitted gaussians for variant functional classification. (A) “Abnormal” (red) and “Normal” (blue) gaussians used to draw thresholds for classifying variants as functionally abnormal or functionally normal. Estimated density from GMM-modeling is on the Y-axis, and SGE score is on the X-axis. Vertical red dashed line at X = -0.0269 represents upper threshold for functionally abnormal variants. Vertical blue dashed line at X = -0.0184 represents lower threshold for functionally normal variants. (B) Strip plot of functional class (Y-axis) vs. SGE score (X-axis)

medRxiv preprint doi: https://doi.org/10.64898/2026.05.15.26352805; this version posted May 21, 2026. The copyright holder for this preprint
(which was not certified by peer review) is the author/funder, who has granted medRxiv a license to display the preprint in perpetuity.
It is made available under a CC-BY 4.0 International license .

Supplemental Figure 2: Pre- and post-workshop survey results on utilizing functional data for variant classification. Breakdown of (A) professional roles and (B) duration of professional experience of workshop attendees (C) Respondents' familiarity with MAVEs (D) Workshop attendees' confidence in using functional evidence for the purpose of variant classification.

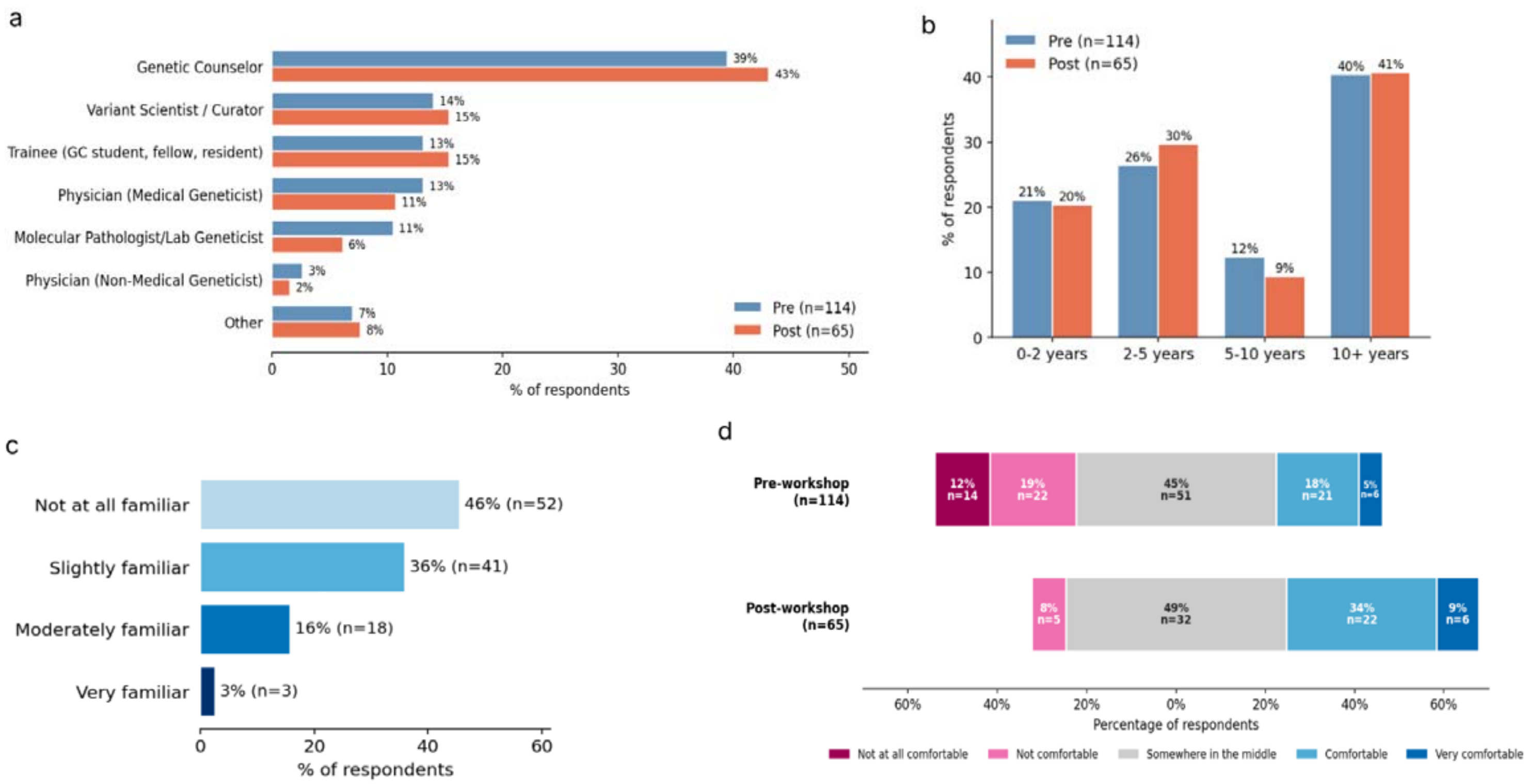

medRxiv preprint doi: https://doi.org/10.64898/2026.05.15.26352805; this version posted May 21, 2026. The copyright holder for this preprint (which was not certified by peer review) is the author/funder, who has granted medRxiv a license to display the preprint in perpetuity.
It is made available under a CC-BY 4.0 International license .

Supplemental note 2: Education workshop slides

Declaration of AI and AI-assisted technologies in the writing process

During the preparation of this work the authors used Claude (Anthropic) in order to proofread and edit the text for clarity and grammar. After using this tool/service, the authors reviewed and edited the content as needed and take full responsibility for the content of the publication.
