# Supplementary material for: Phenotype-Specific Recalibration of MAVE Data Enables Repurposing of BAP1 Functional Assays for Küry-Isidor Syndrome
Source: medRxiv. 2026 May 21:2026.05.15.26352805. Preprint. [Version 1] doi: 10.64898/2026.05.15.26352805 (PMC13228737; doi:10.64898/2026.05.15.26352805)
Supplement: Supplementary file 2 [file media-2.pdf]

Supplementary Table 2. Phenotypic overlap with proband and reported KURIS features

Clinical Feature	KURIS Cohort (n=11) from Kury *et al.* 2023	Present in UDN participant
Global developmental delay	11/11	Yes
Speech/language difficulty	11/11	Yes
Head abnormalities	10/11	Yes (short neck, excess nuchal fold, brachycephaly, midface hypoplasia)
Growth abnormalities	9/11	Yes
Limb abnormalities	7/11	Yes (brachydactyly of fingers and toes, sandal gap, hallux varus)
Musculoskeletal abnormalities	7/11	Yes (decreased peripheral muscle tone)
Seizures	6/11	No
Eye abnormalities	5/10	Yes (bilateral microphthalmia, cataracts, iris dysgenesis, retinal detachments)
Ear abnormalities	4/11	Yes (bilateral high-frequency SNHL; low-set ears; overfolded upper helices)
Cardiac abnormalities	3/10	No
Negative psychiatric disturbances/aggression	3/10	Not assessed
Attention deficits	3/10	Not assessed
Autism spectrum disorder	3/10	Not assessed

*HC= head circumference, SNHL - sensorineural hearing loss
