# Supplementary material for: Phenotype-Specific Recalibration of MAVE Data Enables Repurposing of BAP1 Functional Assays for Küry-Isidor Syndrome
Source: medRxiv. 2026 May 21:2026.05.15.26352805. Preprint. [Version 1] doi: 10.64898/2026.05.15.26352805 (PMC13228737; doi:10.64898/2026.05.15.26352805)
Supplement: Supplementary file 3 [file media-3.pdf]

Clinical Presentation

A pediatric individual was referred to the Undiagnosed Diseases Network (UDN) for evaluation of congenital ocular anomalies, global developmental delay and dysmorphic features. The individual was born via spontaneous vaginal delivery with no complications other than a nuchal umbilical cord requiring brief resuscitation.

In infancy, the individual was noted to have bilateral leukocoria, bilateral microphthalmia, iris dysgenesis, congenital cataracts, and bilateral funnel retinal detachments. Audiologic evaluation confirmed mild bilateral high-frequency sensorineural hearing loss. Dysmorphic features were also noted including a short neck with excess nuchal fold, brachydactyly of the hands and feet, sandal gap, and bilateral hallux varus. The individual exhibited mild to moderate global developmental delay with hypotonia.

Prior Workup

Initial diagnostic workup included a normal chromosomal microarray arr(1-22,X)x2 and negative screen for congenital TORCH infection. Trio exome sequencing with mitochondrial genome analysis identified a *de novo* variant of uncertain significance (VUS) in *AGAP1* (HGNC:16922), NM_014914.5: c.1208T>C (p.Val403Ala). This variant was deprioritized because it was observed twice in healthy individuals (gnomADv4 AF = 2.85-06) and is not known to be associated with human disease.

Workup as part of the UDN

As part of the UDN evaluation, trio genome sequencing was performed on buccal cells which identified a germline *de novo* missense VUS in *BAP1*, NM_004656.4:c.687C>A (p.Asn229Lys).

This variant lies within the UCH catalytic domain of *BAP1*, is absent from population databases, has been reported in ClinVar (Variation ID: 2429331), and has been observed in two other individuals with neurodevelopmental disease (PMID 31785789, ClinVar SCV003799185.3).

Phenotypic Concordance with KURIS

This individual's clinical features substantially overlap with KURIS. Core features present in all reported KURIS individuals, global developmental delay and speech/language difficulty, were evident in this individual (Supplemental Table 2). The ocular phenotype in this individual was notably more severe than previously reported in KURIS. While eye abnormalities have been observed in approximately half of reported individuals, prior descriptions included refractive errors or mild abnormalities. This individual presented with congenital bilateral microphthalmia, cataracts, iris dysgenesis, and bilateral funnel retinal detachments, features not previously described in this syndrome. Features reported in KURIS but absent in this individual include seizures, and cardiac abnormalities. Behavioral and psychiatric features reported in subsets of KURIS individuals, including autism spectrum disorder, attention deficits, and aggression, have not been formally assessed.
